# Clipping of anterior circulation aneurysms using fully endoscopic-assisted minimally invasive keyhole craniotomy: a clinical study and analysis

**DOI:** 10.1007/s10143-025-03226-5

**Published:** 2025-03-08

**Authors:** Huadong Tang, Pengyuan Niu, Dongqi Shao, Shan Xie, Yu Li, Xialin Zheng, Jie Feng, Lei Li, Yuchun Shang, Lulu Chen, Zhiquan Jiang

**Affiliations:** Department of Neurosurgery, The First Affiliated Hospital of Bengbu Medical University, Bengbu, China

**Keywords:** Intracranial aneurysm, Neuroendoscope, Keyhole approach, Subarachnoid hemorrhage, Hunt-Hess grade

## Abstract

Endoscopy’s ability to provide close observation, deep magnification, and multi-angle views has proven to be an effective tool for minimally invasive craniotomy in neurosurgery. However, no large case series have been published on the use of fully endoscopic-assisted minimally invasive keyhole craniotomy for clipping intracranial aneurysms (IAs). To evaluate the value of fully endoscopic-assisted minimally invasive keyhole craniotomy in the treatment of anterior circulation aneurysms. A retrospective analysis was conducted on 20 patients who underwent fully endoscopic-assisted minimally keyhole invasive craniotomy for clipping of IAs. A total of 9 anterior communicating artery (ACoA) aneurysms were clipped using the supraorbital keyhole approach (SKA). Additionally, 10 middle cerebral artery aneurysms (MCA) and 2 posterior communicating artery (PCoA) aneurysms were clipped using the pterional keyhole approach (PKA). The clipping success rate was 100% in all patients. Apart from one patient who experienced transient third cranial nerve palsy, one who developed an intracranial infection, and one who had a brief seizure, no other patients experienced serious complications. Except for one patient who had residual muscle weakness due to a preoperative basal ganglia hemorrhage, all other patients had a modified Rankin Scale (mRS) score of ≤ 1. Fully endoscopic-assisted minimally invasive keyhole craniotomy has promising applications in the treatment of anterior circulation aneurysms in Hunt-Hess grade 0-II, especially for unruptured aneurysms. Future multi-center studies are needed to confirm its broader applicability.

## Introduction

IAs are pathological dilations that occur at major branches of brain arteries [1]. The prevalence of unruptured IAs in the general population is approximately 3.2% [2]. Rupture of these aneurysms can lead to subarachnoid hemorrhage (SAH), which accounts for 80–85% of all non-traumatic SAH cases and 5% of stroke incidents [3]. The treatment of IAs generally involves two main approaches: endovascular coiling and surgical clipping. Since the International Subarachnoid Aneurysm Trial, endovascular coiling has become the primary treatment modality [4]. However, surgical clipping remains indispensable in managing complex aneurysms, such as large or wide-necked aneurysms, aneurysms with branching vessels, those complicated by abnormal vascular anatomy that limits endovascular access, or when complications arise from endovascular procedures that require corrective action [5]. In 1975, Yasagil et al. introduced the classic pterional approach as a standard procedure for treating aneurysms in the anterior circulation [6]. While highly effective, this approach often requires extensive exposure of the skin, bone, and brain, which can increase the risk of iatrogenic complications [7, 8]. In contrast, the concept of “minimally invasive” surgery seeks to reduce unnecessary exposure of the surgical field and minimize the disruption of non-target brain tissue during the operation [9]. In recent years, with advances in neurosurgical techniques, minimally invasive craniotomy has gradually replaced traditional approaches, becoming an important option for treating IAs. The minimally invasive craniotomy techniques for intracranial anterior circulation aneurysms mainly include SKA and PKA [10]. Despite the advantages of minimally invasive techniques, challenges remain, particularly related to the limitations in visibility, which can affect the surgeon’s precision and decision-making during surgery. However, as endoscopic technology continues to evolve, it offers a promising solution to these challenges, improving the feasibility and safety of minimally invasive craniotomy. This study reviews our experience and clinical outcomes with the use of a fully endoscopic-assisted minimally invasive approach for clipping intracranial aneurysms in 20 cases. Through this analysis, we aim to provide valuable insights into the clinical effectiveness of these techniques and offer a reference for the broader adoption of fully endoscopic-assisted minimally invasive approaches in clinical practice.

## Patients and methods

### Patient summary

This retrospective study included 21 anterior circulation aneurysms in 20 patients who underwent fully endoscopic-assisted clipping via SKA or PKA at the Department of Neurosurgery, First Affiliated Hospital of Bengbu Medical University, from January 2023 to June 2023. All patients were diagnosed with intracranial anterior circulation aneurysms through preoperative computed tomography angiography (CTA) or digital subtraction angiography (DSA). Among these patients, 5 (25%) were male and 15 (75%) were female. The mean age of the patients was 63.35 years (SD = 9.11). Of the 21 aneurysms, 10 had ruptured (47.62%), and 11 were unruptured (52.38%). Nine aneurysms were located at the ACoA (42.86%), 10 at the MCA (47.62%), 2 at the PCoA (9.52%). The mean aneurysm size was 6.19 mm (SD = 2.04). Preoperative clinical severity was assessed using the Hunt-Hess grading system: 10 patients were graded as 0 (50%), 4 as grade I (20%), and 6 as grade II (30%). The patients’ condition and basic information are summarized in Table 1.

**Table 1:** Patients summary

Case	Gender	Age	Characteristics of the aneurysms			Hunt–Hess grade	Approach	Time of temporary clipping, minutes	Complications	mRS
			Location	Diameter, (mm)	Ruptured					
1	Female	84	PCoA, L	6.50	Yes	II	PKA, L	6.34	Third nerve palsy	1
2	Female	67	MCA, L	9.40	No	0	PKA, L	/	/	0
3	Female	59	ACoA	3.97	No	0	SKA, R	3.60	/	0
4	Female	75	ACoA	9.00	Yes	I	SKA, R	5.40	/	0
5	Male	60	ACoA	8.10	Yes	I	SKA, L	10.37	Intracranial infection	0
6	Female	63	PCoA, R	4.58	Yes	II	PKA.R	8.53	/	1
7	Female	46	MCA, R	7.10	Yes	II	PKA, R	7.28	/	0
8	Female	50	ACoA	3.32	No	0	SKA, L	4.60	/	0
9	Female	70	ACoA	9.00	Yes	II	SKA, L	6.50	/	2
10	Male	60	MCA, L	8.38	No	0	PKA, L	/	/	0
11	Female	54	ACoA	4.80	Yes	I	SKA, L	5.38	/	0
12	Female	72	MCA, L	2.53	No	0	PKA, B	/	/	0
			MCA, R	4.09	No			3.73	/	
13	Female	53	MCA, R	4.28	No	0	PKA, R	/	/	0
14	Female	70	MCA, R	6.77	No	0	PKA, R	/	/	0
15	Male	62	MCA, L	8.34	Yes	II	PKA, L	5.80	Epilepsy	1
16	Female	67	ACoA	5.12	Yes	II	SKA, R	6.47	/	0
17	Male	70	MCA, L	4.12	No	0	PKA, L	/	/	0
18	Female	71	ACoA	6.92	Yes	I	SKA, L	4.87	/	0
19	Female	59	MCA, R	7.57	No	0	PKA, R	/	/	0
20	Male	55	ACoA	6.15	No	0	SKA, R	/	/	0

*AcoA* anterior communicating artery; *PcoA* posterior communicating artery; *MCA* middle cerebral artery; *L* left; *R* right; *B* bilateral; *PKA* pterional keyhole approach; *SKA* supraorbital keyhole approach; *mRS* modified Rankin Scale

Inclusion criteria for patients: (1) Diagnosis of intracranial anterior circulation aneurysm confirmed by CTA or DSA; (2) Unruptured aneurysms associated with recurrent headaches, cranial nerve palsy, or other related symptoms, or those with a high risk of rupture as determined by evaluation; (3) Ruptured aneurysms with a Hunt-Hess grade of I or II.

Exclusion criteria: (1) Aneurysms larger than 20 mm in diameter or with calcification at the aneurysm neck, which cannot be treated by simple clipping surgery; (2) Aneurysms secondary to infection, trauma, or cerebrovascular malformations, where the underlying conditions may interfere with the study results.

Surgical timing: Emergency surgery was performed for ruptured aneurysms following confirmation of the diagnosis, while unruptured aneurysms underwent elective surgery.

Prior to surgery, a multidisciplinary expert team, including neurosurgeons skilled in endoscopic procedures and those specialized in interventional procedures, assessed the patients’ aneurysm characteristics to determine the most appropriate treatment plans. Then, the team provided the patients with detailed explanations of the advantages, risks, and expected outcomes of both endoscopic clipping and interventional procedures, ensuring that the patients fully understand all available treatment options. The final treatment decisions were made based on the patients’ preferences, with the informed consent forms signed.

### Surgical procedures

Criteria for Approach Selection: For MCA and PCoA aneurysms, the ipsilateral PKA was utilized. For ACoA aneurysms, the SKA was utilized. For SKA, the choice of the left or right approach was determined based on preoperative vascular imaging findings: (1) Dominant A1 Segment: If preoperative angiography revealed a dominant A1 segment (e.g., one A1 segment was significantly larger or the contralateral A1 was hypoplastic or absent), the approach was prioritized on the side of the dominant A1. (2) Frontal Sinus Development: If the diameters of the bilateral A1 segments were comparable, the presence and development of the frontal sinus were considered. The preferred approach was on the side with an absent or smaller frontal sinus. (3) Non-Dominant Hemisphere: If the diameters of the A1 segments and frontal sinus development were similar, the approach was made through the non-dominant hemisphere.

### SKA

**Fig. 1:**
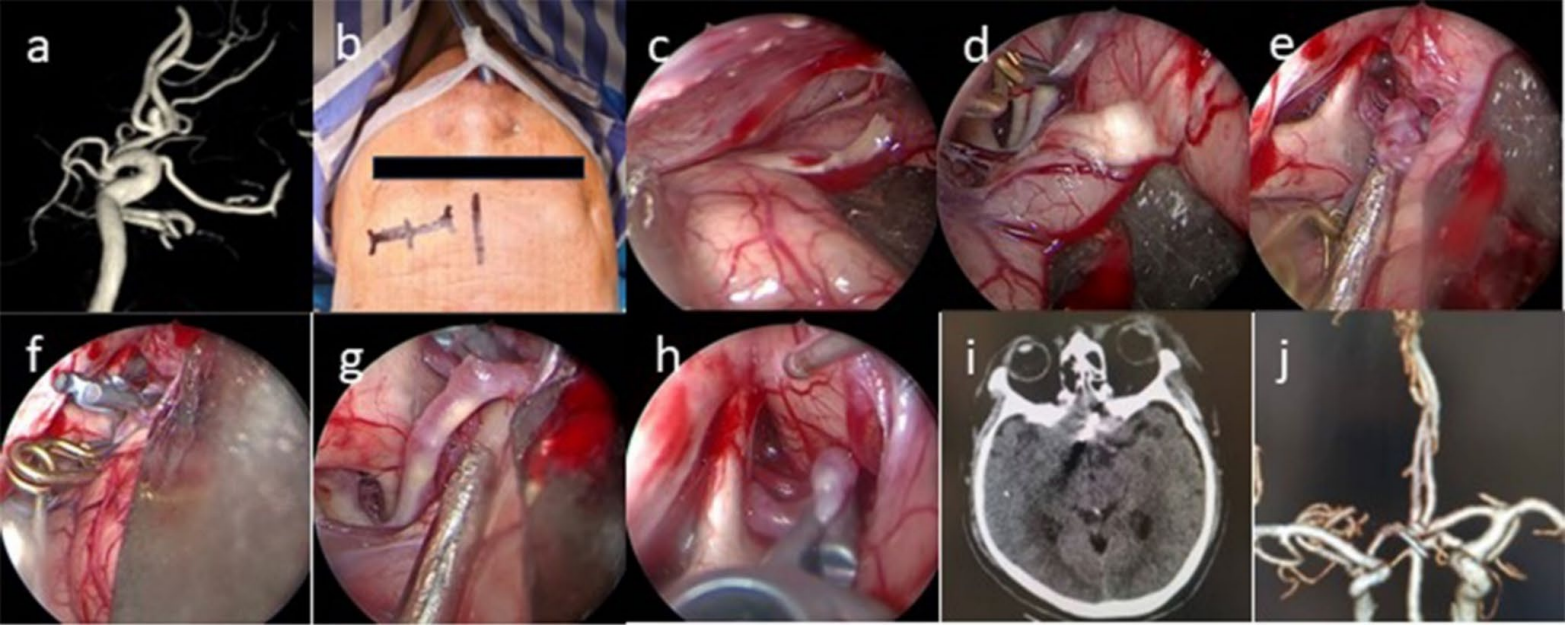
(**a**) Preoperative DSA confirmed the diagnosis of an ACoA aneurysm. (**b**) Due to the patient’s dominant left A1, the left SKA approach was chosen. (**c**) The arachnoid membrane at the base of the anterior cranial fossa was carefully opened, allowing for the release of CSF. (**d**) A temporary occlusion of the A1 segment was performed to facilitate the procedure. (**e**) Distal exploration led to the identification of the aneurysm. (**f**) The aneurysm was successfully clipped. (**g**) Following the clipping, the temporary clip was removed. (**h**) The parent artery and its branches were meticulously examined to ensure proper blood flow. (**i**) Postoperative CT scan showing no bleeding or ischemic changes. (**j**) Postoperative CTA scan showed that aneurysm was clipped completely and the parent artery and its branches were preserved

General anesthesia was administered following orotracheal intubation. The patient was positioned supine, with the head rotated 10°–30° away from the approach to the eyebrow arch and tilted backward 10°–15°. A DORO head brace (Germany) was used to securely fix the patient’s head in place. An arcuate surgical incision, approximately 4 cm in length, was marked along the upper margin of the eyebrow (Fig. 1b). The skin and subcutaneous tissue were carefully incised with a scalpel. Retractors or sutures were employed to laterally retract the skin and underlying muscles. A burr drill was used to create a bone flap, approximately 2–3 cm in diameter. The dura mater was then incised in an arcuate shape. Next, the arachnoid membrane at the base of the anterior cranial fossa was opened, and cerebrospinal fluid (CSF) was released (Fig. 1c). The internal carotid artery and both A1 segments of the anterior cerebral arteries were gently exposed. If preoperative angiography revealed the presence of a dominant A1 or if the aneurysm had already ruptured, temporary occlusion of the A1 was performed before further distal dissection of the aneurysm (Fig. 1d). In the absence of these conditions, the decision to apply a temporary clip was based on the thickness of the aneurysm after careful observation. The dissection continued distally until both the aneurysm and its neck were fully exposed (Fig. 1e). Special attention was paid to any normal vessels or perforators adhering to the posterior surface of the aneurysm or its neck. Once the aneurysm neck was clearly identified, a permanent aneurysm clip was applied to secure the aneurysm (Fig. 1f). A neuroendoscope was then introduced to inspect the aneurysm neck, ensuring proper occlusion, confirming the absence of any residual neck, assessing the occlusion of parent vessels, and verifying that no normal or collateral vessels were inadvertently clipped (Fig. 1h). Finally, the bone flap was repositioned and secured with a titanium plate. The subcutaneous tissue and muscles were sutured with absorbable sutures, and the skin was closed using absorbable subcuticular sutures.

#### Case 8

is presented to illustrate our SKA procedure (Fig. 1).

### PKA

**Fig. 2:**
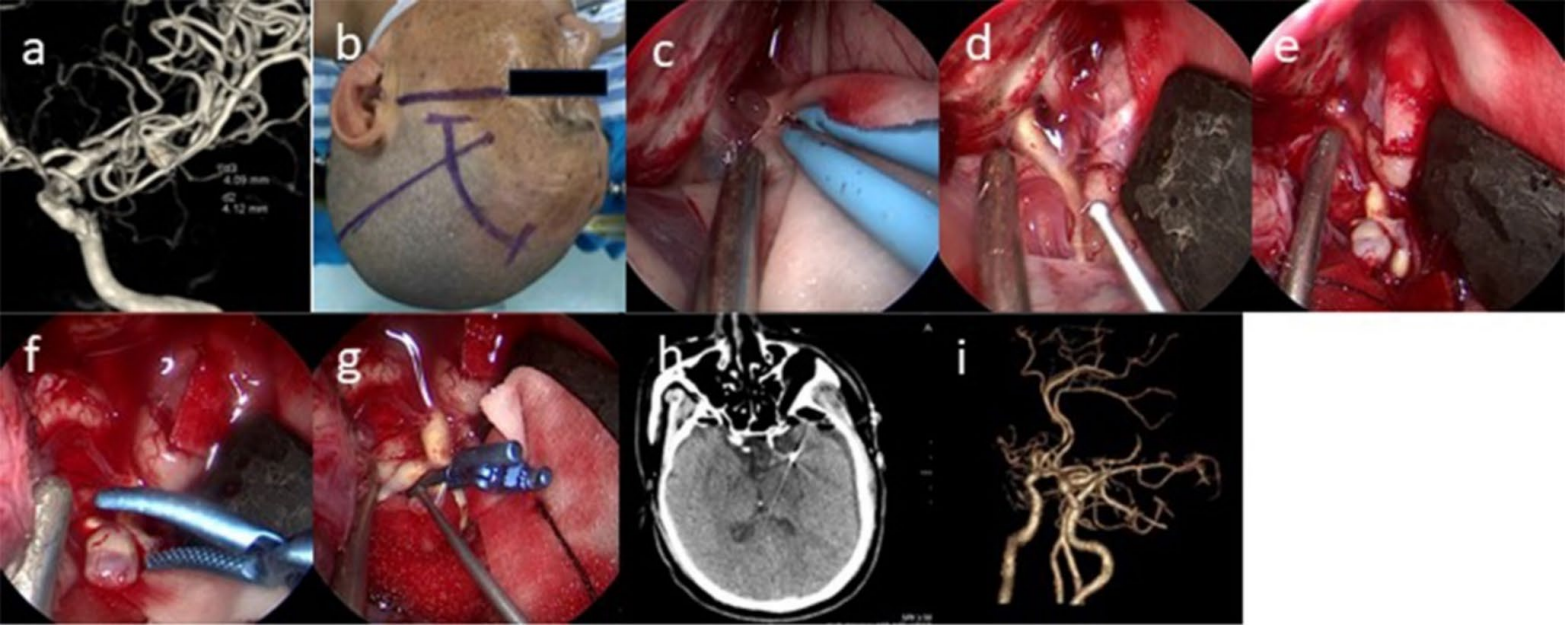
(**a**) Preoperative DSA confirmed the diagnosis of a left MCA aneurysm. (**b**) A PKA was selected for the left side. (**c**) The arachnoid membrane at the Sylvian fissure was opened, and CSF was released. (**d**-**e**) The aneurysm was unruptured, and intraoperative examination revealed a firm aneurysmal sac wall, which did not necessitate the use of a temporary clip. (**f**-**g**) The aneurysm was successfully clipped. (**h**) Postoperative CT scan showing no bleeding or ischemic changes. (**i**) Postoperative CTA scan showed that aneurysm was clipped completely and the parent artery and its branches were preserved

General anesthesia was administered following orotracheal intubation. The patient was positioned supine with the head rotated 10°–30° to the opposite side of the approach to the pterional region. A DORO head brace (Germany) was applied to stabilize the head. An arcuate surgical incision, approximately 7 cm in length, was made along the inner margin of the hairline, centered at the pterion (Fig. 2b). The skin and subcutaneous tissues were incised using a scalpel. Retractors or sutures were employed to laterally retract the skin and muscles. A burr drill was used to create a bone flap, approximately 2–3 cm in diameter. The dura mater was then incised in an arcuate shape. Next, the arachnoid membrane at the Sylvian fissure was opened, and CSF was released (Fig. 2c). The internal carotid artery and M1 segment of the middle cerebral artery were progressively exposed (Fig. 2d). If the aneurysm had ruptured preoperatively, a temporary clip was applied before proceeding with further distal dissection. If the aneurysm was intact, the decision to use a temporary clip was based on careful observation of the aneurysm’s wall thickness. Dissection continued distally until both the aneurysm and its neck were fully exposed (Fig. 2e). Special attention was given to any normal vessels or perforators adhering to the posterior surface of the aneurysm or its neck. Once the aneurysm neck was clearly identified, a permanent aneurysm clip was applied to secure the aneurysm (Fig. 2g). A neuroendoscope was then introduced to inspect the aneurysm neck, ensuring proper occlusion, confirming the absence of residual neck, evaluating the occlusion of parent vessels, and verifying that no normal or collateral vessels had been inadvertently clipped. Finally, the bone flap was repositioned and secured with a titanium plate. The subcutaneous tissues and muscles were sutured with absorbable sutures, and the skin was closed using absorbable subcuticular sutures.

#### Case 17

is presented to illustrate our PKA procedure (Fig. 2).

## Results

In our study, a total of 21 anterior circulation aneurysms in 20 patients were included. Of these, 4 ACoA aneurysms underwent right-side SKA, and 5 ACoA aneurysms were treated with left-side SKA. Among the 9 patients with MCA aneurysms, 1 patient (Case 12) with bilateral middle cerebral artery aneurysms received bilateral PKA, while the remaining cases underwent PKA on the same side. Two patients with PCoA aneurysms received PKA on the same side. All aneurysms were clipped under full endoscopic view. We recorded the usage of temporary clips and the duration of temporary clipping for all aneurysms during the surgery. Temporary clipping was employed in 13 aneurysms (61.90%). The duration of temporary occlusion ranged from 3.60 to 10.37 min, with a median of 5.80 min (interquartile range: 4.87 to 6.50 min). Postoperative complications occurred in 3 cases (15%). One case (Case 5) developed an intracranial infection, which improved following lumbar cistern drainage and antibiotic treatment. One patient (Case 1) experienced oculomotor nerve palsy on the same side post-surgery, and another patient (Case 15) had a brief episode of seizures during hospitalization, which were controlled with valproate, and no further seizures occurred.

All patients underwent a head CT scan on the first day after surgery and had follow-up head CT scans every 7 days before discharge. No hematomas or ischemic events were observed. Follow-up imaging with head CTA was performed 1month post-surgery to assess the aneurysm clipping, and all patients showed no residual aneurysm necks.

All patients will be followed up every 3 to 6 months during the first year after surgery, and the modified Rankin Scale (mRS) was recorded at the final follow-up. Sixteen patients (80%) had an mRS score of 0. Three patients (15%) had a score of 1. One patient (5%) (Case 9) had an mRS score of 2 during the follow-up due to preoperative contralateral limb weakness caused by a basal ganglia hemorrhage.

The selection of the surgical approach, duration of proximal control during surgery, postoperative complications, and mRS scores of the patients are summarized in Table 1.

## Discussion

From a philosophical perspective, the less tissue that is destroyed, the less tissue needs to be healed; the less the brain is exposed, the less damage occurs to the brain; and the smaller the surgical pathway, the fewer tissues and functional structures are at risk [11]. Therefore, smaller craniotomies have long been a goal pursued by neurosurgeons. However, one significant challenge of minimally invasive craniotomies is the reduced visibility and lighting within the surgical field, which may hinder the effective clipping of IAs. For example, perforating arteries or the aneurysm neck extending behind the parent vessel might be overlooked [12]. Even experienced surgeons have reported cases of unintended perforating artery occlusion and incomplete aneurysm neck clipping [13]. Ideally, the best treatment for an aneurysm should be both maximally effective and minimally invasive, with minimal impact on the patient’s quality of life [14]. Compared to a microscope, an endoscope provides better light intensity deep within the surgical field, offers clear, close-up images of pathoanatomical details, and presents a wider viewing angle [15]. These advantages are particularly helpful before, during, and after the microsurgical clipping of IAs, ultimately improving the procedure’s outcome [15]. Because the endoscope can reach deeper within a smaller bony aperture, the required craniotomy may be smaller than that required with traditional microsurgical approaches [16]. Additionally, during the intradural phase of surgery, the magnified and wide surgical view minimizes manipulation or traction of surrounding tissues [17]. In our experience, for some unruptured aneurysms with no perforating arteries or adjacent critical vessels and nerves, aneurysm clips can be applied during surgery through the angled, close-up view provided by the endoscope, even with minimal or no retraction. The flexible adjustment of the endoscope’s rod direction and rotation during the dissection and clipping stages allows surgeons to better expose the aneurysm neck and understand the relationship between the aneurysm, perforating arteries, and nearby blood vessels and nerves [13, 16, 18]. This facilitates timely adjustments to the aneurysm clips. In our study of 20 cases, follow-up neuroimaging results showed no evidence of brain infarction or incomplete clipping of the aneurysm neck, demonstrating the advantages of endoscopy in these procedures.

However, it is important to note that while endoscopy improves the effectiveness of aneurysm clipping, inserting the endoscope into a small cranial aperture can also introduce issues that may not arise with microscope-based techniques, potentially increasing risks and postoperative damage. If a minimally invasive approach results in sub-optimal treatment of the target lesion, it would be unacceptable due to a lack of surgical efficacy [11]. Therefore, the surgical strategy should aim to maximize the advantages of the endoscope while minimizing the potential risks and complications.

One drawback of the fully endoscopic approach is the inability to achieve proximal control of structures outside the scope of the endoscopic lens [16]. To avoid potential damage to structures beyond the endoscopic view, we believe that the choice of approach for aneurysm clipping should follow the principle of “closest route”. The SKA has received significant attention as a minimally invasive technique for lesions in the anterior and middle cranial fossa. Anatomical studies have shown that under endoscopic guidance, structures such as the ipsilateral ICA, A1 and A2 segments of the ACA, M1 segment of the MCA, PCoA, and ophthalmic artery are well exposed and can be easily manipulated. Additionally, the ACoA and medial surfaces of the contralateral ICA, M1 segment of the MCA, and A1 and A2 segments of the ACA are also clearly visible [19]. The value of combining endoscopy with SKA for both ruptured and unruptured ACoA aneurysms has been well established in several studies [20, 21]. Some studies have also used SKA for aneurysms located at the MCA or PCoA positions [22, 23], while we have exclusively employed SKA for ACoA. Following the “closest route” principle, exposure for aneurysms of the MCA or PCoA using a PKA is simpler, with a shorter dissection path, compared to SKA. Moreover, SKA has inherent disadvantages. Firstly, it carries the risk of sinus opening, which can increase the risk of postoperative infection [24]. Although we attempted to minimize or avoid sinus opening when selecting the side for SKA and used gentamicin-soaked gelatin sponge to seal the sinus when sinus opening occured, postoperative infection still occurred in one case. Furthermore, from a cosmetic perspective, while the incision in the facial region is small, it is still visible, and dysfunction of the frontal branch of the facial nerve may complicate cosmetic results [25]. In contrast, the incision in the PKA, which is fully hidden behind the hairline, offers better cosmetic outcomes. More importantly, PKA allows for an easy conversion to a larger craniotomy if necessary [26]. Therefore, for MCA and PCoA aneurysms, even though both SKA and PKA are viable options [27], we tend to prefer PKA. In more complex cases, the principle of " closest route " becomes even more significant. For example, in cases of bilateral MCA aneurysms, the clipping of both aneurysms can be achieved through a single-sided SKA or PKA approach [28, 29]. A unilateral approach avoids the need for a second craniotomy, reduces operation time, shortens hospitalization, lowers treatment costs, and results in better cosmetic outcomes [30]. The challenge, however, arises when treating the contralateral aneurysm. Compared to bilateral craniotomies, even with endoscopic assistance, there is a greater likelihood of residual necks and olfactory dysfunction on the contralateral side postoperatively [31]. Moreover, the endoscope needs to be placed in a deep and narrow working space after extensive dissection of the frontal base, in order to gradually dissect the contralateral M1 segment and aneurysm from the large and tortuous veins in the Sylvian fissure [30]. As the surgical approach becomes deeper, the amount of brain tissue that falls within the endoscope’s blind spot increases, thereby raising the likelihood of iatrogenic damage. The corridor created by extensive dissection of the frontal base significantly amplifies the drawbacks of the endoscope. Therefore, in cases of bilateral middle cerebral artery aneurysms, we opt for a single-stage bilateral craniotomy to separately clip the aneurysms on both sides.

On the other hand, IA surgery often carries the risk of intraoperative rupture, with the incidence rate ranging from 3–50% [32, 33]. Massive bleeding during the operation will block the endoscopic operational view and threaten the safety of the operation. Therefore, whether performing PKA or SKA, it is crucial to achieve effective proximal vascular control before dissecting the aneurysm neck in cases of aneurysms with a high risk of rupture. In our keyhole surgery, we routinely use temporary occlusion clips to control the proximal feeding arteries. This method has proven highly effective for proximal control, and even in the event of an intraoperative rupture, we have successfully avoided converting to microscopic surgery. According to existing reports, the overall usage rate of temporary occlusion clips during aneurysm clipping surgery ranges from 25 to 70%, with the duration of use typically between 2 and 30 min [34–39]. In contrast, in our cases, the frequency of temporary clip usage is higher, while the duration of use is generally shorter. In these keyhole surgeries, to prevent the risk of intraoperative rupture, we routinely achieve proximal vascular control using temporary clips before dissecting the aneurysm neck in cases such as dominant A1 anterior communicating artery aneurysms, ruptured aneurysms, or aneurysms that, although unruptured, have a thin wall detected intraoperatively. As a result, the frequency of temporary clip usage is higher. However, the relatively short duration of temporary clip usage may be attributed to two factors: firstly, our cases are all classified as Hunt-Hess grade 2 or lower, indicating favorable clinical conditions; and secondly, the use of the endoscope provides a broader field of view and more precise anatomical localization, which optimizes the surgical procedure and reduces reliance on temporary clipping [40].

Some studies suggest that endoscope-assisted intracranial aneurysm surgery should be applied to unruptured aneurysms or aneurysms after SAH absorption [13, 41]. In our study, we observed excellent clinical outcomes in patients with unruptured aneurysms, further supporting the significance of endoscopic techniques in these cases. During our preliminary exploration, we performed fully endoscope-assisted keyhole craniotomy exclusively on patients with a Hunt-Hess grade of I or II. Patients with lower Hunt-Hess grades typically experience only mild or no elevated intracranial pressure (ICP), which provides a favorable environment for endoscope insertion [42]. Based on our experience, in these cases, combining gravity with traction, along with appropriately performing endoscopic third ventriculostomy (depending on the condition of hydrocephalus), can create sufficient operative space for the endoscope without the need for additional external ventricular drainage surgery. However, Xie Z and Zhou L have both reported successful outcomes with fully endoscope-assisted keyhole craniotomy for aneurysms in patients with higher Hunt-Hess grades [43, 44]. Therefore, Hunt-Hess grade may not necessarily be a contraindication for employing a fully endoscopic technique in aneurysm surgery. Combining the suggestions of Zhao J [42] with the research findings of Xie Z and Zhou L, we believe that for aneurysms in patients with high Hunt-Hess grades, the decision to use a fully endoscopic approach should depend on the underlying cause of the elevated ICP. If the high ICP is due to severe hydrocephalus, the release of CSF can effectively reduce brain swelling, making fully endoscopic techniques feasible. However, if the high ICP is due to severe brain edema, endoscopic surgery should be considered contraindicated. Clearly, further studies with larger sample sizes are needed to explore the applicability and indications of fully endoscope-assisted keyhole craniotomy in patients with high Hunt-Hess grades.

## Conclusion

Our research suggests that fully endoscopic-assisted minimally invasive keyhole craniotomy has promising applications for the treatment of anterior circulation aneurysms with Hunt-Hess grades 0-II, especially in cases of unruptured aneurysms. When using this technique, it is crucial to adhere to the “closest route” principle and implement effective control of the parent artery to maximize the benefits of endoscopy while minimizing the potential risks and complications. However, this study is based on a retrospective analysis from a single surgeon’s experience with a relatively small sample size, limited to small aneurysms located at common sites typically encountered by neurovascular surgeons. Future multi-center, large-sample studies are needed to further assess the broader applicability of this technique in the treatment of anterior circulation aneurysms.

## Data Availability

No datasets were generated or analysed during the current study.
